# Physician-perceived utility of the EORTC QLQ-GINET21 questionnaire in the treatment of patients with gastrointestinal neuroendocrine tumours: a multicentre, cross-sectional survey (QUALINETS)

**DOI:** 10.1186/s12955-021-01688-x

**Published:** 2021-01-30

**Authors:** Marta Benavent, Javier Sastre, Ignacio García Escobar, Angel Segura, Jaume Capdevila, Alberto Carmona, Isabel Sevilla, Teresa Alonso, Guillermo Crespo, Lourdes García, Neus Canal, Guillermo de la Cruz, Javier Gallego

**Affiliations:** 1 Virgen Del Rocío University Hospital, Biomedicine Institut Biomedicina of Sevilla (IBIS), Av. Manuel Siurot, S/n, 41013 Sevilla, Spain; 2 San Carlos Clinic Hospital, San Carlos Hospital Research Institute (IdISSC), Madrid, Spain; 3 San Pedro De Alcántara Hospital, Cáceres, Spain; 4 Politécnico La Fe University Hospital, Valencia, Spain; 5 Teknon Oncologic Institut (IOT), Teknon Medical Center, Vall Hebron University Hospital, Vall Hebron Institute of Oncology (VHIO), Barcelona, Spain; 6 Morales Meseguer General University Hospital, Murcia, Spain; 7 Clinical and Translational Research in Cancer, Biomedical Research Institut of Malaga (IBIMA), Regional University Hospital and Virgen de la Victoria University Hospital of Málaga, Málaga, Spain; 8 Ramón y Cajal Hospital, Madrid, Spain; 9 Burgos University Hospital, Burgos, Spain; 10 Segovia General Hospital, Segovia, Spain; 11 IQVIA Information S.A., Barcelona, Spain; 12 Ipsen Pharma S.A., Barcelona, Spain; 13 Hospital General Universitario de Elche, Elche, Alicante Spain

**Keywords:** Neuroendocrine tumours, Health-related quality of life, QLQ-GINET21 questionnaire, Communication, Clinical utility

## Abstract

**Background and objective:**

Patient-reported outcome measures can provide clinicians with valuable information to improve doctor-patient communication and inform clinical decision-making. The aim of this study was to evaluate the physician-perceived utility of the QLQ-GINET21 in routine clinical practice in patients with gastrointestinal neuroendocrine tumours (GI-NETs). Secondary aims were to explore the patient, clinician, and/or centre-related variables potentially associated with perceived clinical utility.

**Methods:**

Non-interventional, cross-sectional, multicentre study conducted at 34 hospitals in Spain and Portugal (NCT02853422). Patients diagnosed with GI-NETs completed two health-related quality of life (HRQoL) questionnaires (QLQ-C30, QLQ-GINET21) during a single routine visit. Physicians completed a 14-item ad hoc survey to rate the clinical utility of QLQ-GINET21 on three dimensions: 1)therapeutic and clinical decision-making, 2)doctor-patient communication, 3)questionnaire characteristics.

**Results:**

A total of 199 patients at 34 centres were enrolled by 36 participating clinicians. The highest rated dimension on the QLQ-GINET21 was *questionnaire characteristics* (86.9% of responses indicating “high utility”), followed by *doctor-patient communication* (74.4%), and *therapeutic and clinical decision-making* (65.8%). One physician-related variable (GI-NET patient volume > 30 patients/year) was associated with high clinical utility and two variables (older age/less experience treating GI-NETs) with low clinical utility.

**Conclusions:**

Clinician-perceived clinical utility of QLQ-GINET21 is high. Clinicians valued the instruments’ capacity to provide a better understanding of patient perspectives and to identify the factors that had the largest influence on patient HRQoL.

## Introduction

Neuroendocrine tumours (NETs) are a heterogeneous group of relatively rare neoplasms primarily affecting the lungs, pancreas, and gastrointestinal tract [1]. In most cases, gastrointestinal (GI)-NETs are slow-growing tumours that do not produce clear symptoms until they become metastatic [2–4]. Patients may present a wide range of symptoms, including fatigue, flushing, diarrhoea, dyspnoea, and mood swings, among others, all of which can severely impact health-related quality of life (HRQoL) [5–8]. The main aims of treatment are to control tumour growth, prolong survival [9], and provide symptom relief [7].

In recent years, there has been a growing interest in using patient-reported outcome measures (PROM) to assess patient perspectives to improve care [10, 11]. The use of these instruments to individually manage patients has been shown to improve doctor-patient communication [12, 13] as well as symptom control and HRQoL [12, 14]. The most widely-used PROM for assessing HRQoL in cancer patients is the QLQ-C30, developed by the European Organisation for Research and Treatment of Cancer (EORTC). The condition-specific questionnaire, the EORTC QLQ-GINET21, was developed as an add-on module to the QLQ-C30 to better assess the symptoms and issues most relevant to patients with neuroendocrine tumours [15]. Given the relatively recent development and validation of this instrument (2013), the number of published studies that have used this tool to assess HRQoL in patients with GI-NETs remains limited [7]. Moreover, the perceived clinical value of the QLQ-GINET21 in routine practice has not been assessed to date.

In this context, the main aim of the present study was to evaluate the physician-perceived utility of the QLQ-GINET21 for clinical and therapeutic decision-making in routine clinical practice. A secondary aim was to explore patient-, physician-, and/or centre-related variables potentially associated with the physician-perceived clinical utility of this instrument.

## Material and methods

### Study design

This was an international, observational, non-interventional, cross-sectional, multicentre study conducted in the context of routine clinical practice. In the course of a routine visit, participating patients completed two HRQoL instruments, the QLQ-C30 and QLQ-GINET21, and the participating physicians completed an ad hoc survey designed to gauge their opinion regarding the clinical utility of the QLQ-GINET21.

A total of 36 oncologists and endocrinologists from 34 outpatient clinics in Spain and Portugal participated in the study (NCT02853422). The participating clinics were selected to obtain a representative sample of clinical practice with a wide geographic spread. Reference centres for the treatment of NET in Spain and Portugal were pre-selected and invited to participate (100% agreed to do so). Given that these questionnaires are routinely used in clinical practice and in clinical studies, no specific training in the administration of these instruments was provided.

This study was approved by the Clinical Research Ethics Committee of the Hospital Clínic (Barcelona, Spain). All patients provided written informed consent.

### Patients

Inclusion criteria were: age > 18 years; diagnosis of GI-NET during the past 12 months; ability to complete the study questionnaires and report self-perceived health status. Exclusion criteria were: concomitant participation in another clinical study and presence of any other serious illness.

### Study instruments

#### Clinical utility survey

The participating physicians completed a 14-item ad hoc survey (Table 1) individually for each patient immediately after the study visit. This instrument was designed specifically to assess the physician-perceived utility of the QLQ-GINET21 in routine clinical practice, both overall and on three specific dimensions, as follows: (1) therapeutic and clinical decision-making (7 items); (2) doctor-patient communication (3 items); and (3) questionnaire characteristics (4 items). Each item had five response options, ranging from strongly disagree (0 point) to strongly agree (4 points), with higher scores indicating greater perceived utility. The maximum total score for each dimension varied according to the number of items, as shown in Table 2. Based on the total score on each dimension, the utility was classified as low, moderate, or high (Table 2). This ad hoc survey was not validated as it was specifically designed for the present study.

**Table 1:** Clinical utility survey

*Therapeutic and clinical decision-making*
Item 1. The patient’s responses have helped me to better understand the overall impact of the disease from patient’s point of view
Item 2. The patient’s responses have helped me to better understand the specific aspects/symptoms of the disease that are most limiting or that most affect the patient’s life
Item 3. I have considered the patient’s responses to the questionnaire when selecting the most appropriate treatment
Item 4. I have considered the patient’s responses when making changes in the dose and/or treatment regimen
Item 5. I have considered the patient’s responses when establishing the number and/or frequency of follow-up visits
Item 6. The patient’s responses have helped me to detect other possible health problems and I have referred the patient to other specialists (for example psychiatry)
Item 7. The patient’s responses have helped me to better assess the patient’s current condition
*Doctor-patient communication*
Item 8. The use of this questionnaire improves doctor-patient communication
Item 9. The patient’s responses have allowed me to explore aspects of the disease that I do not usually ask about it during the visit
Item 10. The patient’s responses have helped me to determine whether the patient has received the correct information about his/her disease
*Characteristics of the questionnaire*
Item 11. The instructions are brief and easy for the patient to understand
Item 12. The wording of the questions is easy for the patient to understand
Item 13. The response options are easy to understand and the questionnaire is easy for the patient to fill out
Item 14. The characteristics of the questionnaire enable its routine use in clinical practice

**Table 2:** Characteristics and scoring of the clinical utility survey

Dimension	Score range	Utility classification
Therapeutic and clinical decision-making (7 items)	0–28	Low: 0–9 Moderate:10–19 High: 20–28
Doctor-patient communication (3 items)	0–12	Low: 0–4 Moderate:5–8 High: 9–12
Questionnaire characteristics (4 items)	0–16	Low: 0–5 Moderate: 6–11 High: 12–16

#### QLQ-C30 and QLQ-GINET21

All patients completed, as appropriate, the Spanish or Portuguese versions of these instruments, which were translated according to EORTC translation guidelines [16]. Both scales have been described in detail elsewhere [11, 15]. Briefly, however, the QLQ-C30 is composed of both multi-item scales and single-item measures which include 5 functional scales (cognitive, emotional, physical, role, and social functioning), 3 symptom scales (fatigue, nausea/vomiting, and pain), a global health status/QoL scale, and 5 single items assessing additional symptoms (appetite loss, constipation, diarrhoea, dyspnoea, and sleep disturbance), and perceived financial impact. All scale scores are linearly converted to range from 0 to 100, with higher scores indicating better HRQoL or better function on the relevant scale. By contrast, for individual symptoms and symptom scales, higher scores indicate a higher symptom burden.

The 21-item QLQ-GINET21 questionnaire is administered as an add-on module to the QLQ-C30. This questionnaire includes 4 items to assess muscle and/or bone pain, body image, sexual function, and information. The other 17 items are divided into five scales, as follows: endocrine symptoms (3 items), gastrointestinal symptoms (5 items), treatment-related symptoms (3 items), social functioning (3 items), and disease-related concerns (3 items). Responses are transformed linearly on a scale of 0-100, with higher scores indicating greater symptom severity [15, 17].

The paper-based questionnaires were completed by patients during the study visit. After filling out the questionnaires, the patients returned it to the investigator so that he/she could review the answers and, if necessary, discuss them with the patient. If the patient did not understand a question or how to answer it, the investigator could re-read the question or make minor clarifications, but never interpret the response or answer questions for the patient. Once the visit with the patient had been completed, the investigator transferred the responses from the questionnaires to the corresponding section of the study electronic case report form (eCRF) and filed it (with the patient's consent). The questionnaires were scored during the analysis.

### Study variables

Patient-related sociodemographic variables were: age, sex, employment status, marital status, and educational level. Clinical variables obtained from medical records were as follows: date of GI-NET diagnosis; diagnostic procedure; time since first therapeutic intervention; current medical treatment for GI-NET; treatment modifications (if any) during the visit and reason for change; performance status (European Cooperative Oncology Group, ECOG); symptoms; and concomitant diseases and treatments. The patient’s current health status was assessed by the treating physician and rated as good, neither good nor poor, or poor, according to his/her clinical judgement. In addition, the physician was asked to indicate the patient’s current health status as stable, improving, or worsening in comparison to prior visits. This assessment was based on a general clinical evaluation combined with data, when available, from biochemical, immunohistochemical, and/or imaging studies.

Physician-related variables were age, sex, and years of experience in treating patients with NETs. Centre-related variables were: hospital location (rural, metropolitan, urban) and hospital size (number of beds).

### Outcome measures

The primary outcome measures were the three dimension-specific clinical utility scores obtained on the ad hoc survey, categorized as high, moderate, or low clinical utility.

### Secondary outcome measures

Univariate and multivariate multilevel statistical models were performed to explore the potential association between the clinical utility scores obtained on the ad hoc survey and patient-, physician-, and centre-related study variables.

### Statistical analysis

Descriptive statistics (means, standard deviations [SD], medians, and range) were used for continuous variables. Interquartile ranges were used where appropriate. The number and percentage of patients per response category was used to describe the categorical variables with 95% confidence intervals (CI). Parametric or non-parametric tests were used, as appropriate, to compare means and proportions. Pearson’s correlation (for continuous variables) and ANOVA (for categorical variables) were used to evaluate clinical utility.

Univariate and multilevel regression models were applied to each of the three dimensions to determine whether the variables were significantly associated with the clinical utility of QLQ-GINET21. The multilevel models had two levels, one including the physician- and centre-related variables and the second incorporating patient-related variables. The first model was a null model that did not include any independent variables but rather random intercepts to show the total variance in clinical utility between the centres/physicians. All patient variables with a *P* value < 0.2 on the univariate regression analyses were added to the second model (fixed effects). Finally, the physician- and centre-related variables were added to model 2 to create the third and final model.

Cronbach’s α was calculated for each dimension of the ad hoc survey to determine internal consistency. Values > 0.70 were considered acceptable. Statistical significance was set at p < 0.05. The SAS Statistical software program, v. 9.01 (SAS Institute; Cary, NC, USA) was used to perform all statistical calculations.

## Results

### Physician- and centre-related characteristics

A total of 36 physicians from 34 institutions participated in the study. Sociodemographic data were available for 35 of the 36 physicians (97.2%). The mean (SD) age was 42.7 (7.7) years and 51.4% were female. The mean (SD) years of experience treating NETs was 12.7 (6.7). Nearly all (34/35; 97.1%) of the participating physicians worked in metropolitan/urban areas, most (60.0%) in large hospitals (> 500 beds). Slightly less than half (48.6%) of the physicians treated > 20 NET patients annually.

### Patient characteristics

Of the 209 patients initially recruited, 10 were excluded for failure to meet the inclusion criteria (serious illness [n = 3], failure to complete the QLQ-GINET21 [n = 4], and inclusion after study deadline [n = 3]). Thus, 199 patients (Spain, n = 194; Portugal, n = 5) were included in the final analysis. Of these patients, 92.3% completed both study questionnaires. Table 3 describes the patients’ sociodemographic and clinical characteristics (n = 199).

**Table 3:** Patient sociodemographic and clinical characteristics

Characteristic	Patients^a^
*Age, years, mean (SD)*	61.9 (13.1)
*Sex , n (%)*	
Male	111 (55.8%)
Female	88 (44.2%)
*Marital status (n = 186), n (%)*	
Married/co-habitation	135 (72.6%)
Single	20 (10.8%)
Widowed	18 (9.7%)
Separated/divorced	13 (7.0%)
*Educational leve [(n = 179), n (%)]*	
No formal education	18 (10.1%)
Primary education	68 (38.0%)
Secondary education	52 (29.1%)
University studies/postgraduate studies	41 (22.9%)
*Time elapsed in years since diagnosis of GI-NET , mean (SD)*	2.87 (3.6)
*Time elapsed (years) since the 1 st intervention , mean (SD)*	2.73 (3.6)
*Time elapsed (months) between diagnosis and 1 st intervention , mean (SD)*	1.7 (5.9)
*Pharmacological treatment , n (%)*	
No current pharmacological treatment	67 (33.7%)
Somatostatin analogues	113 (56.8%)
Chemotherapy	21 (10.6%)
Interferon	2 (1.0%)
Targeted therapy	21 (10.6%)
Other	5 (2.5%)
*Presence of symptoms , n (%)*	
No	104 (52.3%)
Yes	95 (47.7%)
*Clinical status according to treating physician*^b^, n (%)	
Stable	151 (75.9%)
Worsening	20 (10.1%)
Improving	28 (14.1%)
*ECOG scores*, n (%)	
ECOG 0	100 (50.3%)
ECOG 1	81 (40.7%)
ECOG 2	16 (8.0%)
ECOG 3	2 (1.0%)
*Presence of concomitant illness*, n (%)	75 (37.7%)
No	
Yes	124 (62.3%)
*Clinical assessment of patient’s health*, n (%)	
Good	169 (84.9%)
Neither good nor poor	9 (4.5%)
Poor	21 (10.6%)

^a^All data, except where indicated otherwise, are based on the full sample (n = 199). In all cases, the percentages are calculated based on the available data

^b^Based on general clinical assessment and, when available, data from biochemical, immunohistochemical, and/or imaging studies

ECOG, European Cooperative Oncology Group; GI-NET, gastrointestinal neuroendocrine tumour; SD, standard deviation

### Clinical utility of QLQ-GINET21 questionnaire

Table 4 shows the mean (SD) scores with 95% CI for the three dimensions assessed on the ad hoc survey.

**Table 4:** Clinical utility of the QLQ-GINET21: results of the ad hoc survey

Dimension	Mean score (SD); 95% CI	Utility
*Therapeutic and clinical decision-making* (7 items)	21.4 (5.8); 20.6–22.2	High
*Doctor-patient communication* (3 items)	9.8 (2.5); 9.4–10.1	High
*Questionnaire characteristics* (4 items)	14.0 (2.1); 13.7–14.3	High

CI, confidence interval; SD, standard deviation

**Fig. 1:**
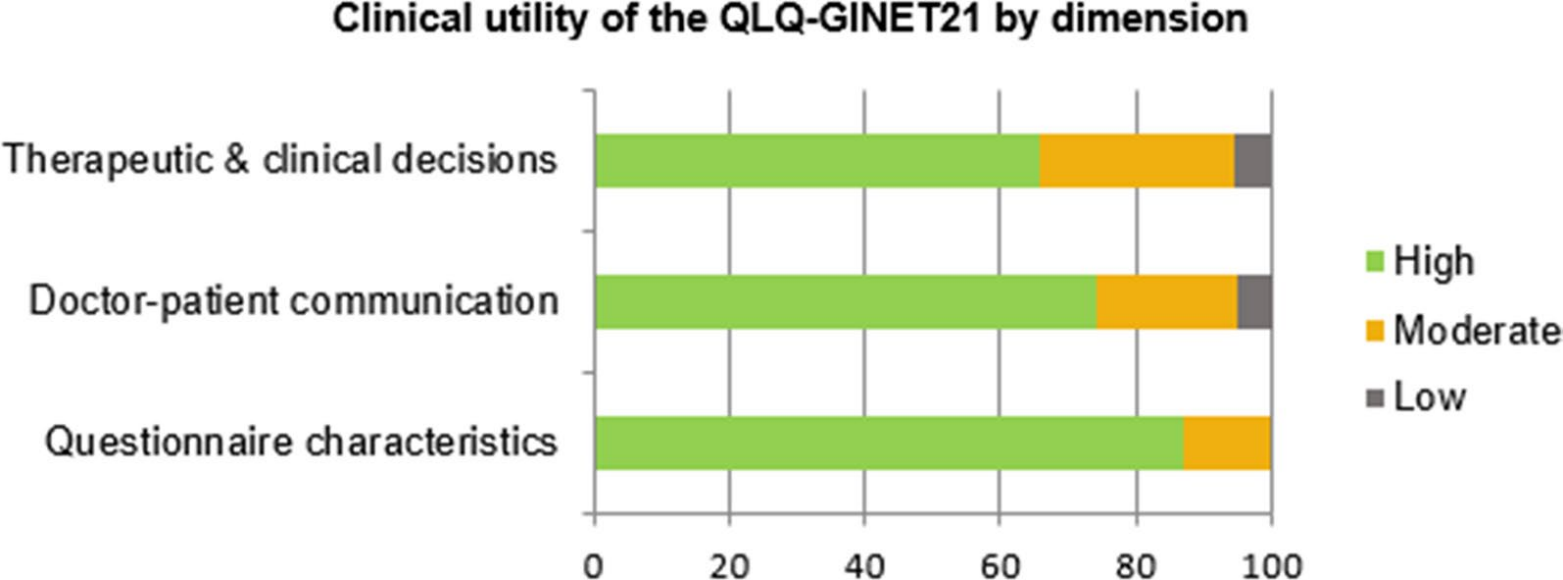
QLQ-GINET21: clinical utility by dimension

Figure 1 shows the percentage of all responses for each dimension categorised as high, moderate, or low utility. The highest-rated dimension was *questionnaire characteristics* while the lowest was *therapeutic and clinical decision-making.*

**Fig. 2:**
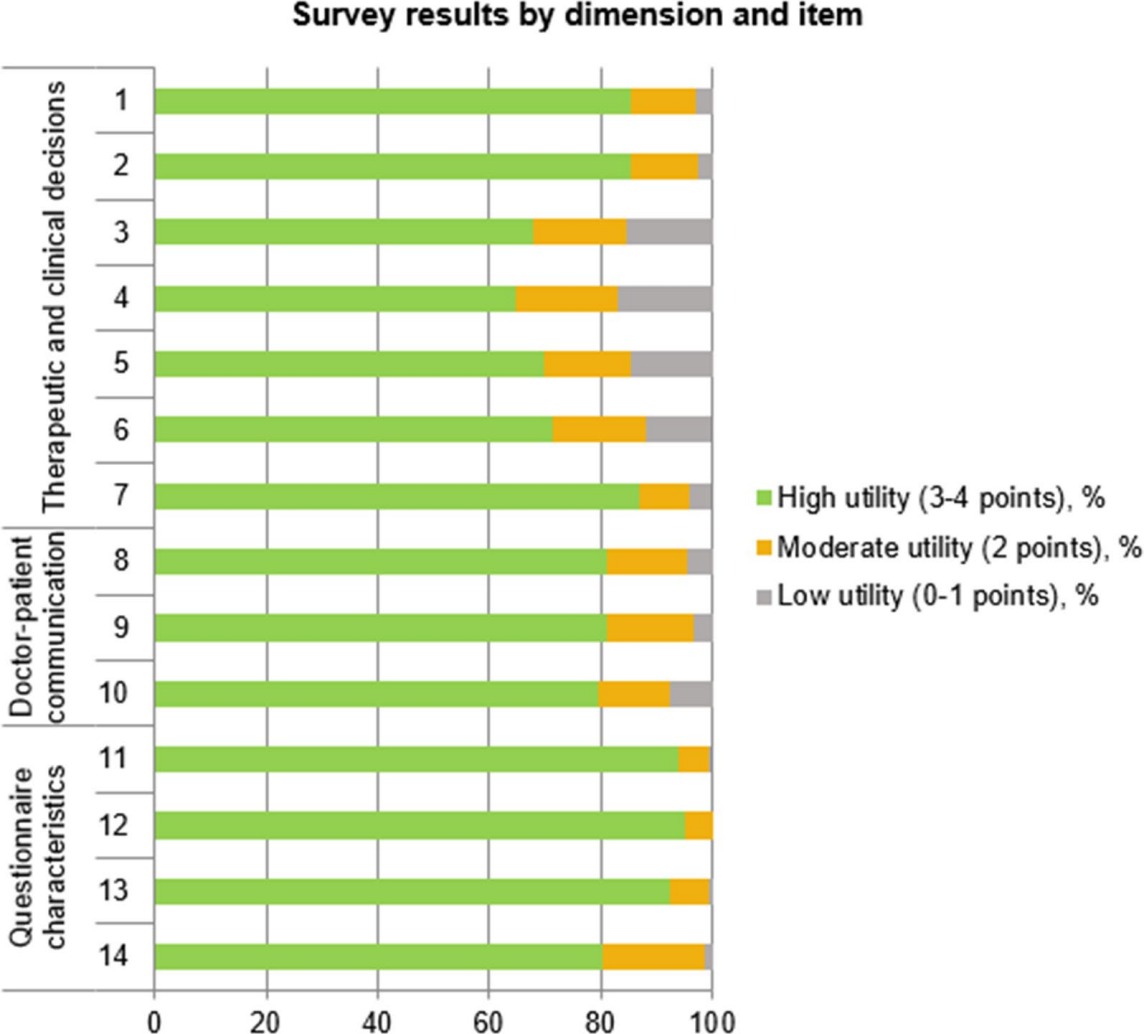
Clinical utility for each item on the ad hoc survey

Figure 2 shows the percentage of responses that rated that item as having high (3–4 points), moderate (2 points), or low clinical utility (0–1 point). As that figure makes clear, the highest rated aspects were items 1, 2, 7, 11, 12, and 13 (high utility on > 85% of responses) while the lowest rated were items 3, 4, 5 (high utility in < 70%). See Table 2 for a description of the individual items.

### Significant associations identified on the univariate analyses

#### Therapeutic and clinical decision-making

The physician- and centre-related variables associated with higher clinical utility scores (mean [SD]) were as follows: female sex, 23.3 (4.6) vs. male sex, 19.9 (6.3), *P* < 0.0001; rural location vs. metropolitan or urban location: 26.4 (3.1) vs. 19.8 (5.8) vs. 23.4 (5.4), *P* < 0.0001; and GI-NET patient volume > 30 patients/year, 23.7 (4.1) vs. 21–30 patients, 17.6 (7.3), *P* < 0.0001. Patient-related variables associated with high clinical utility were good health status: 21.8 (6.0) vs. poor health, 14.3 (4.8); *P* = 0.004; and ECOG 1 performance status, 22.2 (5.0) vs. ECOG 2/3, 17.9 (4.7), *P* = 0.017.

#### Doctor-patient communication

Physician- or centre-related variables associated with higher utility were: female sex, 10.4 (1.9) vs. male sex, 9.2 (2.8), *P* = 0.001; rural location: 11.4 (0.6) vs. urban, 10.3 (2.8) and metropolitan locations, 9.4 (2.3), *P* < 0.001; and hospital size > 200 beds [200–499 beds, 10.0 (2.4) and > 500 beds, 9.7 (2.6)] vs. 100–199 beds, 6.3 (2.5), *P* = 0.030.

Patient-related variables associated with higher utility were no current pharmacological treatment, 10.3 (1.9) vs. current treatment, 9.5 (2.7), *P* = 0.028; ECOG 1 performance status, 10.1 (2.2) vs. ECOG 2/3, 7.8 (2.9), *P* = 0.002; and GI-NET patient volumes: > 30 patients/year, 10.5 (1.5) vs. 21–30 patients, 8.3 (2.9), *P* = 0.002.

#### Questionnaire characteristics

Hospital capacity (> 200 beds) was associated with higher utility (mean [SD]): 200–499 beds, 14.1 (2.0) and > 500 beds, 14.0 (2.2) vs. 100–199 beds, 11.5 (0.6), *P* = 0.043. ECOG 1 performance status was also associated with higher utility: 14.31 (2.1) vs. ECOG 2/3, 13.0 (2.7); *P* = 0.047.

### Multilevel models: significant associations

All patient-related variables that were close to statistical significance (*P* < 0.2) on the univariate regression analyses were entered, together with the significant physician- and centre-related characteristics, in the multilevel models. The results obtained in the final model (model 3) are shown in Table 5 and the key findings for each of the three dimensions are summarized below in Table 6.

**Table 5:** Multilevel model outcomes for patient-related variables^a^

Multilevel model Patient variables								
Therapeutic and clinical decision-making			Doctor-patient communication			Questionnaire characteristics		
Sex								
Male		1.01 (0.50)*	Marital status	Married/cohabitation	0.38 (0.47)	Employment status	Other	0.97 (0.60)
Female		–		Single	– 0.61 (0.59)		Retired	– 0.10 (0.40)
				Widowed	– 0.11 (0.58)		Unemployed	0.25 (0.43)
				Separated/divorced	–		Employed	–
Time since NET diagnosis		0.74 (0.49)	Course of patient’s clinical status	Stable	0.15 (0.35)*	Course of patient’s clinical status	Stable	0.41 (0.32)
				Worsening	0.91 (0.56)*		Worsening	0.76 (0.45)
				Improving	–		Improving	–
Time since first therapeutic intervention		– 0.64 (0.50)	Clinical assessment of patient’s health status	Good	1.96 (0.81)*			
				Neither good nor bad	1.72 (0.79)			
				Poor	–			

^a^Entries show parameter estimate with the standard error in parentheses

*p < 0.05

NET, neuroendocrine tumours

**Table 6:** Multilevel model outcomes for physician- and centre-related variables

Multilevel model				
Physician-/centre-level variables		Therapeutic and clinical decision-making	Doctor-patient communication	Questionnaire characteristics
Age		– 0.09 (0.20)	– 0.23 (0.09)*	– 0.21 (0.08)*
Sex				
Male Female		– 2.61 (1.57) –	– 1.18 (0.72) –	0.42 (0.65) –
Years of experience treating NETs		0.03 (0.23)	0.28 (0.11)*	0.28 (0.10)*
Location of professional activity				
Rural Urban Metropolitan		9.20 (4.26)* 4.51 (1.54)* –	0.85 (1.93) 0.78 (0.70) –	1.16 (1.92) – 0.39 (0.64) –
Hospital capacity				
100–199 beds 200–499 beds ≥ 500 beds		4.30 (4.40) – 0.64 (1.61) –	– 1.50 (2.01) 0.97 (0.74) –	– 0.49 (1.84) 0.78 (0.67) –
Number of patients with NETs treated per year				
0–10 pts/year 11–20 pts/year 21–30 pts/year > 30 pts/year		– 3.14 (2.14) – 4.75 (2.24)* – 6.47 (2.13)* –	– 0.17 (0.97) – 1.65 (1.02) – 2.22 (0.97)* –	– 0.55 (0.89) 0.50 (0.93) – 0.87 (0.88) –

Entries show parameter estimates with standard errors in parentheses

*p < 0.05

Estimation method = ML; Satterthwaite degrees of freedom. NET, neuroendocrine tumours

#### Therapeutic and clinical decision-making

Only one patient-related variable (male sex) was significantly associated with higher utility. High patient volumes (> 30 GI-NET patients/year) were associated with higher utility. By contrast, a metropolitan work location was associated with lower utility.

#### Doctor-patient communication

Physician-perceived clinical utility of the QLQ-GINET21 was significantly lower in patients with an “improving” health status vs. stable/worsening and for those with “poor” versus “good” health status. Two physician-related variables (older age and less experience in treating patients with NETs) were significantly associated (*P* < 0.05) with lower clinical utility on this dimension.

#### Questionnaire characteristics

Two physician-related variables (older age and less experience in treating patients with NETs) were both significantly associated (*P* < 0.05) with lower clinical utility on this dimension.

#### Internal consistency of the ad hoc survey

To determine the internal consistency of the scale, we calculated the Cronbach’s α for all three dimensions *therapeutic and clinical decision-making, doctor-patient communication,* and *questionnaire characteristics*), with values > 0.80 on all three dimensions (respectively, 0.807, 0.898 and 0.908), thereby indicating good reliability.

## Discussion

The main objective of the present study was to assess physician perspectives regarding the clinical value of administering the QLQ-GINET21 in routine clinical practice. Overall, our findings show that the clinicians surveyed considered this instrument to have a high clinical utility (Figs. 1, 2). The highest-rated dimension was *questionnaire characteristics*, followed by *doctor-patient communication*, and finally *therapeutic and clinical decision-making*. The highest-rated items on the survey (Fig. 2, Table 1) were items 1, 2, 7, 11, 12, and 13 (rated “high utility” in > 85% of cases) while the lowest rated items were items 3, 4, 5 (< 70% of “high utility” ratings). On the multilevel model, one physician-related parameter (GI-NET patient volumes > 30/year) was associated with high clinical utility for *therapeutic and clinical decision-making*. Two physician-related variables (older age and less experience treating GI-NETs) were associated with low clinical utility on two dimensions (*doctor-patient communication* and *questionnaire characteristics)*. Overall, these findings confirm that physicians find the QLQ-GINET21 to be useful to help inform clinical practice.

The results of this clinician survey show that these professionals highly valued the brevity, clarity, and ease of administration of the QLQ-GINET21, characteristics that allow for rapid administration in the clinic, which allows them to provide patients with immediate feedback, thus improving doctor-patient communication. Our findings suggest that the main clinical utility of the instrument from the physicians’ perspective is that it provides a better global understanding of patient perspectives, particularly those factors that most impact HRQoL, including aspects that may otherwise be overlooked.

There is a large and growing body of literature[18–21] on the role and benefits of condition-specific PROMs, showing that the routine use of such instruments can improve communication between doctors and their patients and help with clinical decision-making. The findings of the present study confirm these benefits for the QLQ-GINET21. While some clinicians may be reluctant to systematically administer PROMs due to concerns about the associated increase in workload and/or misconceptions about the utility of such instruments [22], assessing patient perspectives has become increasingly relevant, particularly in patients with GI-NETs, who have a relatively long life expectancy and for whom quality of life is a key outcome measure [23].

### Therapeutic and clinical decision-making

The dimension that received the lowest clinical utility ratings (considered “high utility” in only 65.8% of cases) was therapeutic and clinical decision-making. However, if we group the “high” and “moderate” utility scores, this percentage rises to approximately 95%, indicating an overall moderate-to-high support for the clinical utility of the QLQ-GINET21 for clinical decision-making. The relatively lower perceived utility of this dimension (Fig. 1) versus the other dimensions is not surprising given that this dimension comprises the most complex aspects of patient care, and patient-reported data are only one piece of the complex clinical picture that the clinician must consider. Moreover, the capacity of this instrument to help improve clinical decision-making is limited by the fact that the minimal clinical important differences (MCID) for this instrument have yet to be defined[23].

As Fig. 2 shows, the items with the greatest perceived clinical utility in this dimension were the items that help to provide a better understanding of the patient’s perspective regarding the impact of the disease (item 1), the aspects that most impact QoL (item 2), and current disease status (item 7). The relatively high clinical utility of these items suggest that physicians are interested in obtaining a greater understanding of the patient’s perspective. By contrast, the items with the lowest perceived utility (all of which received a score of 3 or 4 in < 70% of cases) were items 3 (treatment selection), 4 (changing dose or treatment regimen), and 5 (establishing frequency/number of follow-up visits).

### Doctor-patient communication

The three items on this dimension all received similar utility ratings (around 80%), indicating that, in general, the physicians considered the questionnaire to be of value in improving communication. Item 8 on this dimension assesses the value of the questionnaire to improve doctor-patient communication in general. Item 9 refers to the value of the QLQ-GINET21 to explore overlooked aspects of the disease. Item 10 refers to the value of the questionnaire to help determine whether the patient has received enough information about the disease. Overall, the findings on this dimension suggest that the QLQ-GINET21 may help to improve doctor-patient communication. a finding that is in line with previous reports regarding the QLQ-C30 and similar instruments [18–20].

### Questionnaire characteristics

This dimension, which broadly assesses the design and ease of use of the QLQ-GINET21, was the most highly rated, indicating that the physicians in this survey believe that the questionnaire is sufficiently brief and easy to understand, clearly-worded, and easy to complete. Moreover, these results are supported by the high completion rate (92.3%) of the QLQ-GINET21 in our cohort, a finding that is consistent with the 90% completion rates reported by Yadegarfar et al. in the validation study for this instrument [15]. Notably, the lowest-rated item (80.4%) in this dimension was item 14 “the characteristics of the questionnaire enable its routine use in clinical practice”, indicating that some physicians believe there are impediments to using the questionnaire in routine practice. In this regard, the QLQ-GINET21 is generally administered together with the QLQ-C30, thus increasing the time needed for completion. Indeed, a pilot study conducted by Van Leeuwaarde et al.[24] to assess patient (rather than physician) perceptions of the QLQ-GINET21 found that 13% of the respondents considered the questionnaire either too long or too tiring. This finding, considered together with our results, suggests that the instrument could potentially benefit from further refinement, such as removing the least valued aspects (as rated by physicians and/or patients) to reduce the time needed to complete the QLQ-GINET21. For example, in our survey, 7.5% of respondents indicated that item 10 (which asks whether patients have received enough information about the disease) had little clinical utility. Despite the proven utility of HRQoL questionnaires, their use in clinical practice is sometimes limited due to time limitations [25], which may be particularly relevant in patients with GI-NETs in whom fatigue is a common symptom.

A secondary, exploratory aim of this study was to identify patient-, physician-, or centre-related variables that might be associated with perceived clinical utility. On the multilevel model, high NET patient volume (> 30 patients/year) was associated with higher perceived utility on the *therapeutic and clinical decision-making* dimension, perhaps because physicians who treat more patients have greater experience in using and interpreting the results of the QLQ-GINET21. Conceivably, as physicians become more familiar with the instrument, this could increase their ability to use it more effectively and interpret it more quickly. Two other physician-related variables (older age and less experience) were associated with low clinical utility on two dimensions (*doctor-patient communication* and *questionnaire characteristics*). The association between older physician age and lower clinical utility is consistent with previous reports [26, 27]. However, in our study, this finding appears to contradict our other finding that less experience in treating NETs was also associated with low clinical utility. These contradictory findings are difficult to explain but may be related to the relatively small number of participating physicians (n = 36). The routine use of PROMs in clinical practice has increased substantially in the past two decades. Numerous studies have demonstrated the value of these instruments to inform care [28] and to improve communication [12, 13], symptom control, and HRQoL [12, 14]. Such instruments are particularly valuable in chronic diseases with a high symptom burden such as GI-NETs. However, the development and validation of these instruments is a complex, time-consuming process that requiring an ongoing evaluation process through field-use and studies such as ours. The QLQ-GINET21 is a relatively new instrument and it is imperative that we continue to refine it for the benefit of both patients and clinicians alike. Studies such as ours and that by Van Leeuwaarde et al. [29] can provide valuable data that can be used to improve these questionnaires. For instance, those authors found that while most (83%) of the patients who completed the survey considered that the instrument provided a moderate (30%) or good (53%) indication of their HRQoL, nearly one in five (18%) patients considered it a “poor” representation of their HRQoL. These findings, considered together with the results of our study, underscore the importance of assessing PROMs such as the QLQ-GINET21 from the perspective of both clinicians and patients.

### Study strengths and limitations

This study has several limitations. First, the number of participating centres and clinicians was relatively small, making it difficult to draw statistically significant conclusions, especially regarding to the physician- and centre-related variables. In addition, this was a cross-sectional study with only a single HRQoL assessment per patient.

Another limitation is that the QLQ-GINET21 was designed for use at an aggregate level, rather than for individual patients. Since the MCID for this instrument have not been defined and validated[23], this could have impeded the clinician’s ability to accurately interpret the items, potentially leading the participants to overrate the true utility of certain items. By contrast, the main strength of this study is that it is the first to assess the perspective of physicians regarding the clinical utility of the QLQ-GINET21 in routine clinical practice.

## Conclusions

The findings of this study show that the participating physicians consider the QLQ-GINET21 to have a high clinical utility. The most highly-rated aspect was the characteristics of the questionnaire design (brevity, comprehensibility, and ease of administration). For clinical decision-making, the most highly-rated facets of the QLQ-GINET21 were its capacity to provide a general understanding of patient perspectives and, more specifically, the disease-related aspects with the greatest impact on patient HRQoL.

## Data Availability

Where patient data can be anonymised, Ipsen will share all individual participant data that underlie the results reported in this article with qualified researchers who provide a valid research question. Study documents, such as the study protocol and clinical study report, are not always available. Proposals should be submitted to DataSharing@Ipsen.com and will be assessed by a scientific review board. Data are available beginning 6 months and ending 5 years after publication; after this time, only raw data may be available.

## Abbreviations

CI: Confidence interval
ECOG: European Cooperative Oncology Group
eCRF: Electronic case report form
EORTC: European Organisation for Research and Treatment of Cancer
GI: Gastrointestinal
GI-NET: Gastrointestinal neuroendocrine tumours
HRQoL: Health-related quality of life
NET: Neuroendocrine tumours
SD: Standard deviation
